# Comparison of Two Different Feather Sampling Methods to Measure Corticosterone in Wild Greater Flamingos (*Phoenicopterus roseus*) and Wild Mallards (*Anas platyrhynchos*)

**DOI:** 10.3390/ani11102796

**Published:** 2021-09-25

**Authors:** Marielu Voit, Katrin Baumgartner, Lorenzo von Fersen, Roswitha Merle, Lukas Reese, Mechthild Wiegard, Hermann Will, Oriol Tallo-Parra, Annaïs Carbajal, Manel Lopez-Bejar, Christa Thöne-Reineke

**Affiliations:** 1 Institute for Animal Welfare, Animal Behavior and Laboratory Animal Science, Freie Universität Berlin, Königsweg 67, 14163 Berlin, Germany; Mechthild.Wiegard@fu-berlin.de (M.W.); Christa.Thoene-Reineke@fu-berlin.de (C.T.-R.); 2 Zoo Nuremberg, Am Tiergarten 30, 90480 Nuremberg, Germany; Katrin.Baumgartner@stadt.nuernberg.de (K.B.); lorenzo@vonfersen.org (L.v.F.); Hermann.Will@stadt.nuernberg.de (H.W.); 3 Institute for Veterinary Epidemiology and Biostatistics, Freie Universität Berlin, Königsweg 67, 14163 Berlin, Germany; Roswitha.Merle@fu-berlin.de; 4 Zoologischer Stadtgarten Karlsruhe, Ettlinger Straße 6, 76137 Karlsruhe, Germany; lukas.reese@zoo.karlsruhe.de; 5 Veterinary Faculty, Universitat Autònoma de Barcelona, Campus UAB, 08193 Bellaterra, Spain; Oriol.Tallo@uab.cat (O.T.-P.); anais.carbajal@uab.cat (A.C.); Manel.Lopez.Bejar@uab.cat (M.L.-B.); 6 College of Veterinary Medicine, Western University of Health Sciences, Pomona, CA 91766, USA

**Keywords:** feather corticosterone, Mallard, Greater Flamingo, wild birds, comparative study, cut feathers, plucked feathers, less invasive, animal welfare, refinement, sex comparison

## Abstract

**Simple Summary:**

The common standard sampling method to determine corticosterone in feathers (CORTf) is to pluck them from the bird’s skin. This procedure is considered to be painful, and the animals have to be caught and fixated firmly. Therefore, an animal experiment approval is required according to European and German legislation. In this study, we compared two methods: plucking vs. cutting feathers. The aim was to confirm the validation of an alternative less-invasive sampling technique. The specimens of this project were wild adult Mallards (Germany) and wild 1st-calender-year juvenile Greater Flamingos (Spain). In summary, there were no significant differences between the methods in terms of corticosterone results for both species. Additionally, no differences were found in CORTf between females and males of both species. In conclusion, these findings underline the suitability of cutting feathers as a sampling method for the determination of CORTf levels.

**Abstract:**

This research project had the aim to validate the possible alternative and less-painful sampling method of cutting feathers close to the skin instead of plucking them for subsequent feather corticosterone analysis, confirming recently-published results for other species in captivity. Analyzing CORTf is often used in animal welfare studies in combination with behavioral monitoring. The background of this idea was to act in the sense of animal welfare and reduce the burden of animal studies according to the 3-R-Principle (Replacement, Reduction, and Refinement) by refining procedures. To confirm the hypothesis that the sampling method itself has no influence on CORTf levels measured, plucked and cut samples of the respective bird were collected. Birds of two wild species were used: the Mallard (*Anas platyrhynchos*) and the Greater Flamingo (*Phoenicopterus roseus*). The CORTf was measured by using an enzyme-linked immunosorbent assay (ELISA). The determined values were inspected for their mean values, standard deviation (SD), and average differences. Afterwards, the CORTf levels of both species were compared, according to the sampling method, with the concordance correlation coefficient (CCC). In the Bland-Altman (BA) plot the differences of the methods were displayed against the mean values. Additionally, sex, as a possible factor influencing CORTf, was analyzed using the Mann-Whitney U test. The values of CCC showed poor agreement in the comparability of the two methods, whereas the concordance of the BA plot was decent. The average differences between the methods were marginal for both species (Mallards: −0.16 pg/mm, Flamingos −0.13 pg/mm). In summary, all anomalies or differences between the methods were negligible. Therefore, the alternative sampling method seems to be as suitable as the common standard method. No significant difference was found between females and males. Nevertheless, our results suggest that CORTf should not be interpreted in just considering the values themselves, but the results they should be analyzed in the context of a wider set of parameters. Hence, further studies are encouraged to create a larger data pool.

## 1. Introduction

To assess the well-being of a bird, different approaches have been developed so far. One possible way to evaluate the animal’s welfare is to combine behavioral observations with corticosterone (CORT) measurements. Corticosterone is the main glucocorticoid (GC) in birds, reptiles, amphibians, and rodents, whereas in other mammals and fish, it is cortisol [1,2]. An increasing level of GC is especially associated to the presence of stress stimuli [3,4]. In the case of a sudden stimulus, the individual first experiences an increased secretion of catecholamines, followed by activation of the hypothalamic-pituitary-adrenal (HPA) axis and the release of GC into the bloodstream (fight-or-flight response) [4,5]. This response has diverse effects on the bird’s physiology (e.g., increasing heartrate and blood pressure) and helps to reduce risk and ensure survival [4,6]. However, GC cannot only be seen as a ‘stress-hormone’ [5]. It is released in baseline concentrations, as well as in various concentrations which can be related to different internal and external influencing factors [2]. Such influencing factors could be age, sex, or body condition, and it varies with climate or season, for instance [2]. To interpret the GC levels, it is important to retrace the actions of the HPA axis and understand the reactions to chronic stress. Sapolsky et al. have described the GC impacts as preparative, stimulating, suppressive, or permissive to maintain homeostasis in challenging occasions [4]. In other studies, the concept of allostasis has been used as a basis, a situation in which stability is preserved through change [7,8]; Romero et al. have modified it in their ‘reactive scope model’ (integrating homeostasis, allostasis, and stress) [9].

Nowadays, measuring corticosterone in feathers (CORTf) is an increasingly used and validated method to evaluate stress levels [3]. The CORTf enables the researcher to obtain a retrospective view of the activity of the bird’s HPA axis during the time of feather growth [3,10]. In the molting period, blood circulates in the feather follicles, and CORT diffuses into the feather. As described in a study by Bortolotti et al. and then confirmed in other studies, increased plasma CORT levels during feather growth result in elevated CORTf levels [11,12,13,14]. To determine CORTf, the feathers are sampled by plucking them from live or dead birds or even by collecting the ones dropped by molting birds [10,11,13,15]. Depending on the scientific context, the aspect of a long-term and retrospective view on the activity of the HPA axis could be a clear benefit compared to other corticosterone measurement methods. For good sample quality, feather growth should be completed at the time of sampling, and the feather should be dry and clean; only then, the CORTf level can be related to the time of feather growth [10]. An advantage of CORTf measurement is the storage of feathers. Because feathers are a stable matrix, except of storing them dry and clean, there they require no further treatment [3,16,17,18,19]. This enables longer storage, and thus, e.g., samples of rare species or wild birds can be collected when the opportunity arises and analyzed at a later time if needed.

In a previously published study, we addressed the issue of validating an alternative method, cutting feathers, in captive geese and ducks instead of feather plucking, the common sampling method, for CORTf measurement [20]. In addition, we evaluated those results in this study with wild birds and dealt with the influencing factor ‘sex’. The reason to validate an alternative method was that the plucking of a feather is regarded painful for the bird because it results in reactions of the body, e.g., increasing heart rate and blood pressure or behavioral changes, which may be associated with pain [21,22,23,24]. Therefore, to examine CORTf in plucked feathers of living birds, an animal experiment application is needed in Germany and many other EU countries. To act in the sense of animal welfare and according to the EU Directive 2010/63/EU and 3-R Principle, with the aim to reduce animal distress, the idea of the alternative and less-invasive method of feather-cutting arose [25,26], where the feather can be cut off near the bird’s skin. As in a previous study [20], the region between the shoulders was chosen as sampling site in this study. The localization was perfect for fast and safe sampling and had no impact on the bird’s flight ability. This sampling region has already been used in other research projects [19,27,28]. An additional point in favor of the alternative cutting technique is that in the process of CORTf determination, the calamus of the feather is first cut off before the feather is analyzed in more detail (e.g., measurement of length and weight) [11,16,28,29]. Consequently, plucking the feather is not necessary for the following analysis.

In this research project, two species of wild animals were examined: adult Mallards (*Anas platyrhynchos*) and 1st-calender-year juvenile Greater Flamingos (*Phoenicopterus roseus*). The wild Mallards were shot for hunting reasons, whereby no animal experiment was needed. Regarding the flamingos, through the project “Anillamiento de flamencos”, we had the opportunity to sample wild Greater Flamingos [30]. The application for the animal experiment has already been approved by the competent authorities in Spain [31,32]. With these chosen wild species we had the chance to discuss the comparability of CORTf to results of two other studies. Drawing a comparison of the wild ducks of this study with the Mulard Ducks (*Anas sterilis resp. Cairina moschata × Anas platyrhynchos*) of a conventional poultry farm was possible [20]. Additionally, the availability of feathers from juvenile flamingos allowed a comparison with recent study results on adults [27]. The German Veterinary Association for Animal Protection (Tierärztliche Vereinigung für Tierschutz—TVT) investigates and evaluates husbandries of certain captive bird species ethologically and physiologically in more detail [33]. Both orders of Anseriformes and Phoenopteriformes have been listed by the TVT to encourage science-based animal welfare assessments.

The aim of this study was to validate cutting as a less-invasive method to determine CORTf in wild bird species and to confirm existing results that suggest the sampling method itself has no effect on the measured CORTf levels [20]. In addition, a comparison of CORTf between female and male birds was examined to consider sex as a possible influencing factor. Furthermore, an outlook was formulated on the comparability of data between captive and wild birds of this study.

## 2. Materials and Methods

### 2.1. Sampling Protocol

The feathers were plucked and cut close to the skin between the shoulders, as described before [20,27,34,35,36]. The wearing of gloves while sampling avoided any contamination (e.g., sweat), and additionally, the researcher ensured that the feather quills were dry and free of any biological contamination (e.g., blood, feces). All samples had the same morphology (cover feathers) and a similar total length (300–400 mm). In addition, to obtain a better agreement in the comparison of CORTf levels, the selected feathers had the same color within the species (flamingos with gray feathers, Mallards with brown feathers) [10,37,38,39]. A minimum sample size of 45 animals of each species was determined biometrically, resulting in an equivalence test of means with 90% power and 5.0% significance level, whereas the true difference between the means was 0.0 [20]. The samples were stored in paper envelops at room temperature until laboratory examination. Another possible influencing factor of CORTf levels was included: the samples were differentiated according to the individual’s sex [3,11,36,37,40]. An ethical review did not need to be approved in this study because the ducks were hunted and the sampling of flamingos was conducted in the context of an already approved project in Spain [30].

### 2.2. Specimens

First, it should be made clear that the most accurate information possible on a bird’s habits, molting status, and biological data is crucial for interpreting CORTf [3,17]. However, since this study was based on wild birds, no individual data were available. Therefore, the focus was placed on the knowledge about the populations of the respective species.

#### 2.2.1. Mallard (*Anas platyrhynchos*)

The Mallard is assigned to the genus group of dabbling ducks (*Anatinae*) [41,42]. They are widespread and common in the Eurasian and North American regions as well as in parts of North Africa [43]. The duck species sampled in this study is the native and most common of its species in Germany: *Anas platyrhynchos platyrhynchos*. This species is not water-bound, but they stay on the water after sunrise for resting and dabbling [42]. Their habitat is near to water, such as lakes, small ponds, rivers, and in winter also on the seashore [44]. One of the peculiarities of ducks is the beak structure, which is similar to that of the flamingo. The lamellae of the beak and the tongue create a kind of sieve apparatus that filters food such as small animals, insects, or green plant parts from the water [41]. In general, they are omnivores. The courtship of the ducks occurs in fall, passes into an engagement period, the fixed mating in January/February, and, finally, the egg-laying period in March, followed by a breeding period of around 28 days [42]. The ducklings are nidifugous, covered in down, but are able to eat, swim, and dive [45]. They reach flight maturity in summer at the same time as the adult drakes after their large plumage molt in June or July [41]. The general molt of the duck takes place later, at the end of July and the beginning of August [42]. The small-feather molting occurs twice a year [41]. In October, the drake changes to its splendor feather dress for the mating season, which it keeps until spring [42]. In this period, the animals show apparent sexual dimorphism whereby they are distinguishable visibly.

The hunting season for Mallards in Germany is legally set from 1 September to 15 January [42,44]. The sampling took place on several days from 27 October until 28 December 2019 after the specimens were shot in hunting manners in the district “Nürnberger Land”, east of Nuremberg. Both sexes and the ducklings of the same year are capable of flight at this time, therefore it is important to distinguish them on the basis of certain characteristics: The drakes wear their unique plumage for the mating season, which made it possible to differentiate the sexes visibly. In Mallards, juveniles can be recognized by their darker, gray-greenish feet and reddish horn-colored bill, whereas adults have light yellow feet, which even turn to orange-red from the second year of life [42].

In total 47 Mallards, 22 females and 25 males (differentiated via visible sexual dimorphism), were sampled. All specimens were identified as adults (via visible characteristics). A minimum of 10 feathers were sampled by plucking as well as by cutting them closely to the skin from each duck.

#### 2.2.2. Greater Flamingo (*Phoenicopterus roseus*)

The Greater Flamingo is classified in the order of *Phoenicopteriformes* [41]; its distribution area is in Africa, Asia, and Europe [46]. The flamingos of this study were located in southwest Spain in Marismas del Odiel, a nature reserve nearby Huelva.

Flamingos live together in large flocks at salt lakes, salt pans, coastal brackish waters, or similar ecosystems [41]. In this habitat, there is often mass reproduction of the brine shrimp (*Artemia salina*), which, together with copepods and insect larvae, is on the food list of flamingoes, followed by algae and diatoms [41,45]. Similar to the Mallards described above, the flamingo has a unique beak that is used in combination with the tongue to filter these brine shrimp; however, the beak is inverted during filtering, so the mandible is uppermost [45]. It is very difficult to differentiate male and female Greater Flamingos [41]. In Spain, the breeding period usually lasts from April until June [47]. The classification of the study specimens in terms of their age was done following the definitions of Grzimek’s Animal Life Encyclopedia [41]: egg incubation = 27–31 days; precocial = 4–7 days after hatching; white-gray down, red feet, and red, straight beak = newborn until 7–10 days; black feet and black, straight beak = after age of 7–10 days; molting in second gray down and bill bending = age of 2–3 weeks; small plumage in shoulder area = age of 4 weeks; flight ability = age of 70 days; juvenile plumage = gray-brown; molting in pale plumage = age of 9–18 months; full coloration = age of 3–4 years.

The specimens were sampled in combination with the project “Anillamiento de flamencos” on 19 July 2019. This project is organized and supported by several Spanish and European organizations and has been existing since 1986 [31]. Sampling takes place annually in two natural reserves in Andalusia, Marismas del Odiel, and Fuente de Piedra, where large colonies of Greater Flamingos breed. In this operation, the colony was slowly circled in the early morning hours by many volunteers. The adult flamingos flew away, leaving the chicks that were still unable to fly. After caging them, each chick was individually captured, weighed, photographically documented, sampled for blood and feathers, ringed, and finally released back into the wild. These annual examinations and documentations serve to monitor the colony and its reproductive success [32]. With the official approval of the whole project by the Spanish authorities, feather sampling on living flamingos was allowed for our study. The chicks already had a curved beak and were still in a gray plumage, which, however, had molted to the first small plumage in the shoulder area. Considering the Spanish breeding season, the age was around 4 weeks to less than around 10 weeks old, since the flamingos were still unable to fly.

A total number of 46 juvenile flamingos were sampled. Per sampling technique (cutting and plucking), 7 to 10 feathers were collected. Since the juvenile birds could not be distinguished externally with certainty in terms of sex, feathers had to be examined separately for each bird by DNA analysis (laboratory of ‘Tauros Diagnostik—Veterinärmedizinische Analysen’). It turned out that 26 female and 20 male flamingos were sampled.

### 2.3. Analysis of CORTf

The measurement of CORTf was performed with an ELISA kit. For feather processing, we strictly followed the protocol of Bortolotti et al. (2008) and the modified version of Monclús et al. (2017) [11,16]. To obtain accurate results, it is additionally recommended to perform a new assay validation for each species under investigation [29].

First, several feathers from both Mallards and flamingos were tested to determine the minimum total feather length needed to produce sufficient powder for the measurement and the optimal time in the ball mill to obtain a fine and uniform powder. For both species, a length of 300 to 400 mm was determined per sampling method, which resulted in 9 to 10 feathers required for each Mallard and 7 to 10 feathers for each Greater Flamingo. In addition, to avoid confounding factors, the sampled feathers needed to be from the same type and, thus, morphologically identical. The first step of further processing is cutting off the calamus in the plucked samples [11,16,29]. Additionally, it was ensured that the calamus was completely off at the cut feather samples; otherwise, it was touched up. The feather samples were weighed to the nearest of 0.1 mg, and subsequently, the ball mill (Retsch^®^, MM200 type with two balls and 25 Hz, Germany) ground the samples to a particle size of ~10 µm for 4 min for the relatively small feathers. The weight of the powder was compared to the starting weight to check for major loss. Next, the feather powder of each sample was mixed with 1.5 mL of methanol in a vortex (Vortex Mixer S0200–230 V-EU; Labnet International, Edison, NJ, USA) at room temperature for 30 min, and the mixture was incubated at 37 °C for 18 h in a G24 Environmental Incubation Shaker (New Brunswick Scientific, Edison, NJ, USA). Hereafter, the sample was centrifuged at 3500× *g* for 15 min (Hermle Z300K; Hermle^®^ Labortechnik, Wehingen, Germany). From the resulting supernatant, 1 mL was pipetted into an Eppendorf^®^ tube, which was then placed in an oven (Heraeus Function Line T6^®^, Thermo Fisher Scientific, Waltham, MA, USA) at 38 °C until all the liquid had dried. The residue was dissolved in 0.25 mL of the buffer solution (containing BSA, NaCl, EDTA and Azide) of the commercial enzyme immunoassay kit (ELISA Neogen^®^ Corporation, Ayr, UK) and mixed in the vortex for 1 min. If the sample was not immediately used in the ELISA for CORT measurement, it was frozen at −20 °C until analysis.

### 2.4. Statistical Analysis

The precision of the ELISA was verified by the inter- and intra-assay coefficient of variation (CV) [48,49,50]. For measuring the CV, a pool of 10 different samples of 10 different individuals was created to run the assay in triplicates, whereas the actual samples were analyzed in single runs. We evaluated the intra- and inter-assay CV using a pool with both species, resulting in an intra- and inter-assay CV of 9.44 and 11.96%, respectively. The same kit and protocol have previously been validated separately for both species, with similar results [20,27].

All samples were in good condition and not contaminated with blood or feces; therefore, all CORTf results could be used for analysis [50]. Corticosterone values were expressed as pg CORTf/mm feather length. Since it can be assumed that the feather growth rate is uniform in morphologically similar feathers of one individual and sampling region, hormone exposure is time-dependent and should therefore be specified by length [3,11,17,18,51].

Data were collected in MS Excel^®^ version 2016 and analyzed with IBM SPSS v. 25.

To assess the agreement between plucked and cut feathers, the CORTf of each method was compared within the same individual. For this, Lin’s concordance correlation coefficient (CCC) and the Bland-Altman plot (BA-plot) were used for analysis.

**Figure 1:**
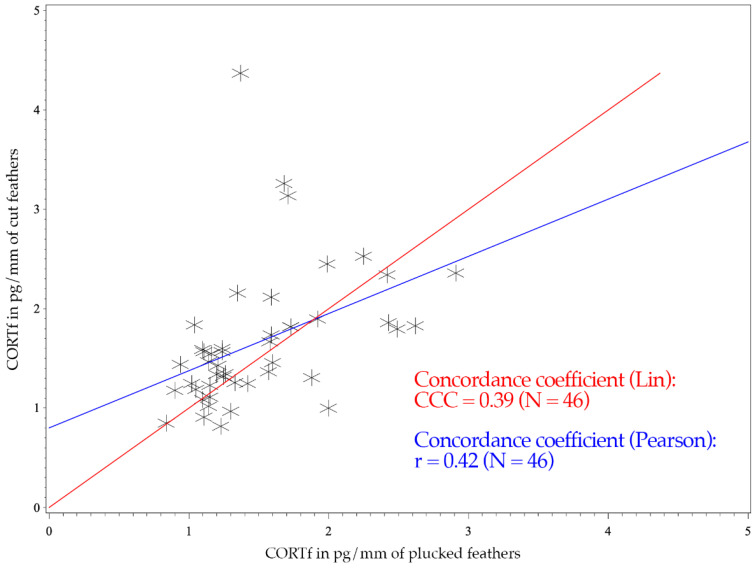
CCC and Pearson’s concordance coefficients of 46 Mallards (*Anas platyrhynchos*). The *x*-axis shows the CORTf values of plucked feathers in pg/mm, and the *y*-axis represents the values of cut feathers in pg/mm. The red line illustrates Lin’s concordance coefficient. Pearson’s correlation coefficient is expressed with the blue line.

**Figure 2:**
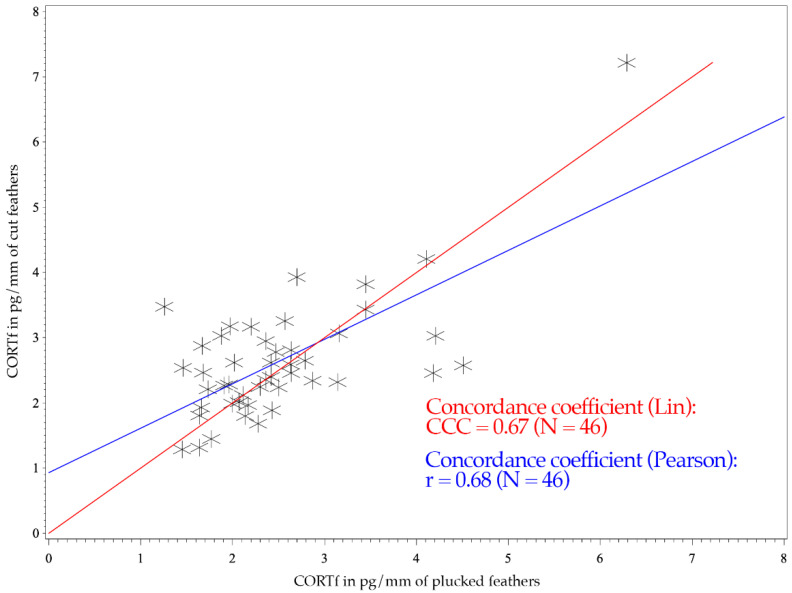
CCC and Pearson’s concordance coefficients of 46 Greater Flamingos (*Phoenicopterus roseus*). The *x*-axis shows the CORTf values of plucked feathers in pg/mm, and the *y*-axis represents the values of cut feathers in pg/mm. The red line illustrates Lin’s concordance coefficient. Pearson’s correlation coefficient is expressed with the blue line.

The figure of the CCC presents the degree of dependence of the observed values to the 45° line through the origin, indicating perfect agreement (see Figure 1 and Figure 2) [52]. Consequently, the CCC measures the precision and accuracy of a test method [53].

**Figure 3:**
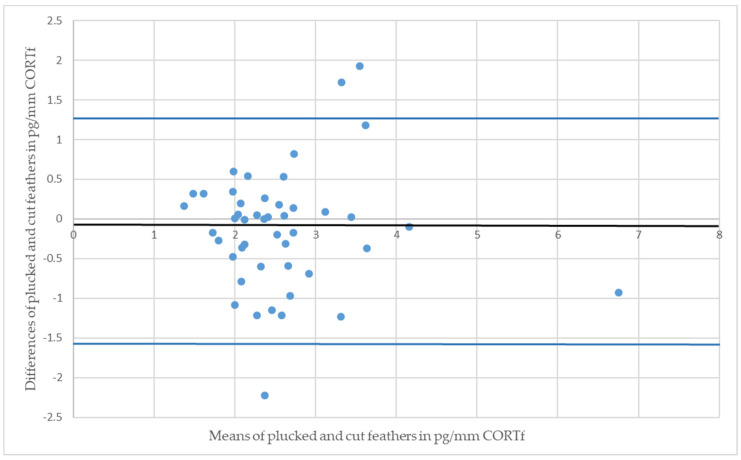
Bland-Altman plot of Mallards (*Anas platyrhynchos*); the residual plot displays the differences (*y*-axis) against the means of values (*x*-axis) of CORTf in the two sampling methods. The blue horizontal lines represent the mean difference ± 2 SD (mean = −0.16, SD = 0.65).

**Figure 4:**
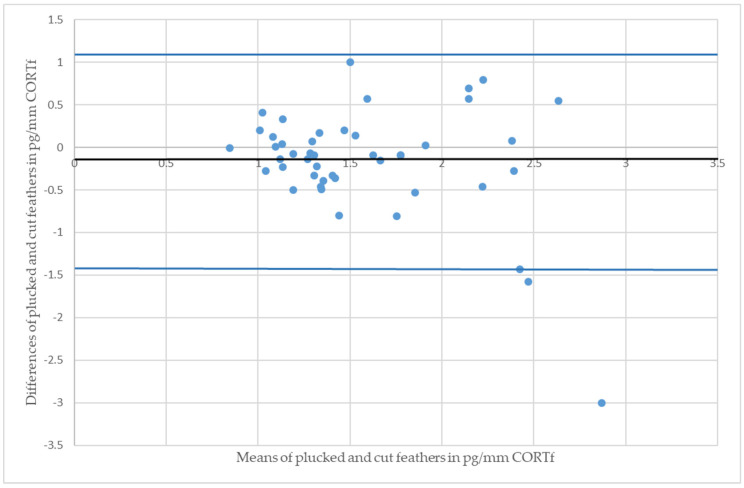
Bland-Altman plot of Greater Flamingos (*Phoenicopterus roseus*); the residual plot displays the differences (*y*-axis) against the means of values (*x*-axis) of CORTf in the two sampling methods. The blue horizontal lines represent the mean difference ± 2 SD (mean = −0.13, SD = 0.76).

Regarding the figures of the BA-plot, the difference between CORTf of the two sampling techniques of one individual were set in relation with the respective means of values (see Figure 3 and Figure 4). Each point represents a pair of values in the graph; the centerline is the mean difference, complemented by a 95% confidence interval. The image of points should not show an ascending or descending tendency, but scatter around the mean difference. Subsequently, the BA-plot assess the agreement between the two sampling methods and displays differences and outliers [20,54,55].

**Figure 5:**
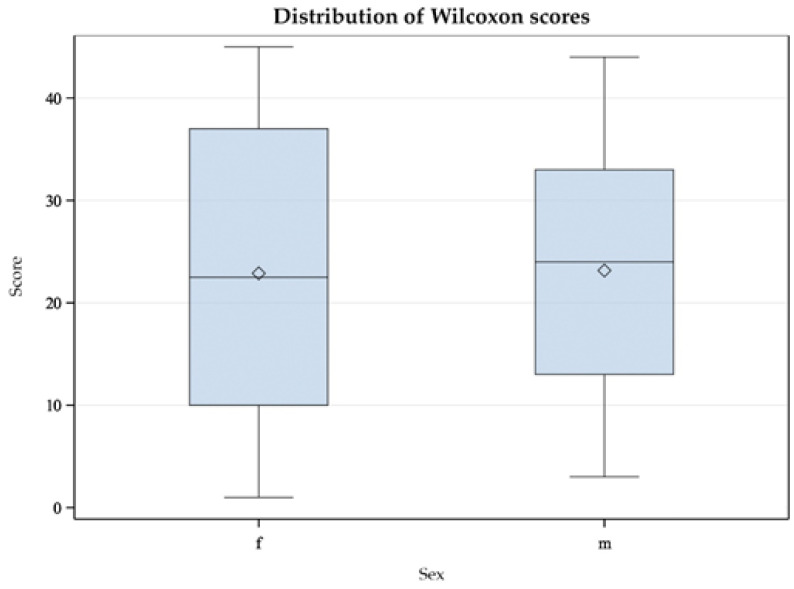
Mann-Whitney U test with double-sided t-approximation plotting the differences of female (f) and male (m) Mallards (*Anas platyrhynchos*) in their CORTf levels.

**Figure 6:**
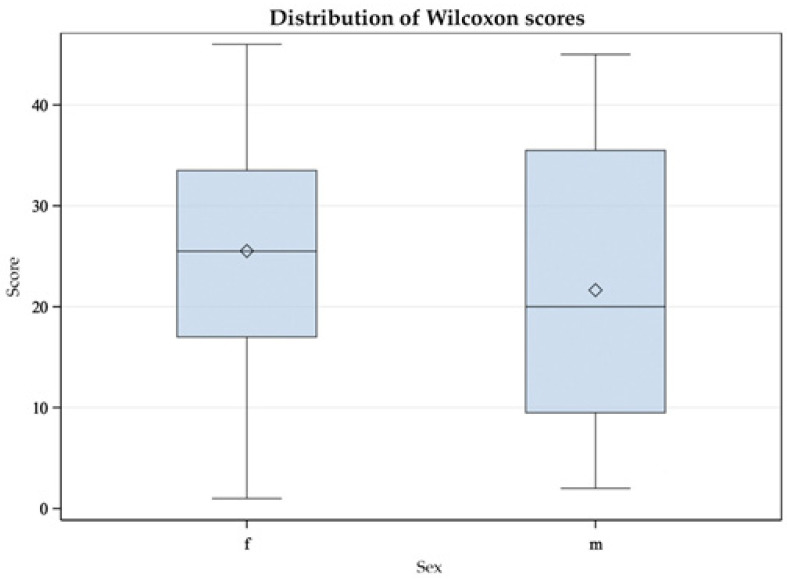
Mann-Whitney U test with double sided t-approximation plotting the differences between female (f) and male (m) Greater Flamingos (*Phoenicopterus roseus*) in their CORTf levels.

In similar studies with Common Redpolls (*Acanthis flammea*) and Snow Petrels (*Pagodroma nivea*), a difference in plasma CORT level between the sexes was found [37,40]. Therefore, we investigated the relationship between sex and CORTf level using the Mann-Whitney U Test. This test was applied because the data were not normally distributed [56]. Results are displayed in a box-plot figure (see Figure 5 and Figure 6).

## 3. Results

In the following analysis, we had a total number of 47 Mallards, with 22 females and 25 males. The CORTf value of one male was identified as an outlier (8.10 pg/mm in the cut samples), and this individual was therefore excluded from analysis (see Table 1). This resulted in a total of 46 ducks. This number was equal to the number of sampled flamingos (*n* = 46), although we examined 26 females and 20 males (see Table 2). In the results for the Greater Flamingos, one value did not correspond to the specified norm: the minimum total length of plucked feathers was 236 mm. Thus, the value fell below the requirement for total length. Equally notable is the significantly higher (>2 pg/mm) maximum value of CORTf in pg/mm in female flamingos compared to males, irrespective of the sampling method. In total, the feather samples of the Mallards compared to the Greater Flamingos had a higher total length, by 18 mm (plucked) and 14 mm (cut), and an overall lower CORTf level, by 1.01 pg/mm (plucked) and 0.97 pg/mm(cut), respectively. An overview of the CORTf levels of both species, their other feather characteristics (length, weight), and a comparison of the results between the two sampling techniques, as well as between sexes, is displayed in Table 1 and Table 2.

The Mallards had a CCC of 0.39 and a Pearson’s concordance coefficient of r = 0.42 (see Figure 1); whereas the measurements of Greater Flamingos resulted in a CCC value of 0.67 and a Pearson’s concordance coefficient of r = 0.68 (see Figure 2). Considering the BA-plot, Mallards had an average difference of all CORTf levels in pg/mm between the two methods of −0.16. They produced a standard deviation of 0.65, with a 95%-confidence interval ranging from +1.12 to −1.44. The CORTf values of Greater Flamingos created an average difference of −0.13, with a standard deviation of 0.76, resulting in a 95%-confidence interval from +1.36 to −1.61.

The differences in CORTf levels between female and male Mallards were not significant, with *Z* = 0.97, *p* = 0.3384 (see Figure 5). There was also no statistically significant difference in CORTf between sexes in Greater Flamingos, with *Z* = 0.06, *p* = 0.9544 (see Figure 6).

## 4. Discussion

Overall, the samples were in very good condition, without any contamination, which allowed us to comply with the required minimum number of 45 individuals for both bird species. This provides a good basis for drawing meaningful conclusions from the results.

The results of this study show no relevant differences in CORTf according to the different sampling techniques, namely plucked versus cut feathers. Thus, our results confirm the findings of our previous study in captive Domestic Geese (*Anser anser domesticus*) and Mulard Ducks (*Cairina moschata × Anas platyrhynchos resp. Anas sterilis*) [20]. Likewise, the sex of the birds did not appear to have any influence on the CORTf level. Nevertheless, there are a few findings and aspects of the study that need to be discussed below.

In this study, as in two of our previous studies, we tried to standardize the samples as much as possible [20,27]. This was not only related to the living conditions and biological data of the sampled animal, but fundamentally related to the sampled feathers. We aimed to achieve standardization by choosing the same localization: feathers were both cut and plucked between the shoulders of each individual. Furthermore, we ensured that the morphology of the feathers to be examined was the same. Finally, the CORTf level was also standardized over the total length of the feathers. It must be added here, however, that perfect standardization of the samples can only be achieved by precise identification of the collected feathers, for example, by sampling the first and second wing feathers [20]. However, as we want to use this method in future field studies with airworthy birds, it is important to find sample locations where feathers can be taken without affecting the airworthiness of the bird. Therefore, we think that feathers from the region between the shoulders are better suited, even if the standardization may be slightly impaired. This region has already been selected for CORTf measurements in Greater Flamingos [27], Broilers (*Gallus gallus domesticus*) [28], Red Kites (*Milvus milvus*) [19], Domestic Geese and Mulard Ducks [20].

The inter- and intra-assay CV values of the pool of both species were below the reference values, and the performance of the assay was considered acceptable. Whereby, the results of CORTf had a good reliability and repeatability.

Regarding the CORTf levels of the Mallards, one individual produced a value of 1.13 pg/mm in the plucked feather sample, whereas its cut sample resulted in 8.10 pg/mm. This result was considered an outlier because no values above 5 pg/mm were found in the whole group of ducks. In addition, the other sample of this individual did not create such high values. Such an outlier could have been caused by measurement errors, and therefore, this individual was excluded from the analysis, and the total examined number of Mallards was reduced to 46. The maximum CORTf value was higher in cut feathers, and the minimum value was higher for plucked feathers, which led to a higher range of CORTf levels with the cut sampling technique (see Table 1). In total, the cut samples produced slightly higher average values. However, these differences were marginal and therefore interpreted as negligible. Regarding the Greater Flamingos, no outlier was observed, but one individual produced high (>5 pg/mm) CORTf values with both removal techniques. It generated a level of 6.29 pg/mm with plucked feathers and 7.22 pg/mm with cut feathers. These were by far the highest maximum values in the group of flamingos, and we assume that this individual had an elevated CORT level in the plasma at the time of feather growth, which could be interpreted as some form of stress. If these values were excluded, the total average value would be decreased by 0.15 pg/mm. However, since these values were plausible, they were not removed. As described earlier, the overall CORTf levels of Mallards were lower compared to those of flamingos. The Greater Flamingos were still in their gray juvenile plumage. A larger variation of CORT levels in plasma has been described in some studies in young birds in contrast to adults [57,58]. However, the results for the flamingos clustered as expected, and the average difference between the two methods was not large at −0.13 pg/mm, even when compared to the Mallards (−0.16 pg/mm).

The CCC of both species were regarded as poor, with a worse mismatch for the Mallards (0.39 vs. 0.67). However, regarding the figures, the values clustered as desired and showed a good agreement (see Figure 1 and Figure 2). In Figure 1 (Mallards), three individuals stood out, which had relatively high cut CORTf levels (>3 pg/mm), but plucked values that only ranged between 1 and 2 pg/mm. In the case of flamingos, the overall values were higher, but they produced a good match (see Figure 2). Nevertheless, four points were remarkable: two results with high plucked values (>4 pg/mm) and, in addition, low cut values (<3 pg/mm), a high cut CORTf level (>3 pg/mm) with a low plucked value (1–2 pg/mm) and an individual which created both very high plucked and cut CORTf levels. For these few values, the discrepancy between the two methods was increased. In summary, the poor CCC should not be viewed too critically since we had a good agreement in the comparison of the CORTf values within each individual between the methods, resulting in a basically coherent graph. The few results with the higher discrepancy were exceptions, which might had occurred because we were not comparing the identical feather (e.g., first wing feather) or because of an error in sample processing. In addition, blood quills dry at different times, even if they grow close together, which may also cause minimal differences in CORTf as a result of a slightly shifted time frame of feather growth. However, in general, regarding the whole group of birds, these differences are not significant.

For both species, Pearson’s correlation coefficient was r > 0. A positive correlation implies that higher values of cut samples (*y*-axis) were associated with higher values of plucked ones (*x*-axis) (see Figure 1 and Figure 2). Focusing on the BA-plot, the standard deviation of Mallards was 0.65, which was lower than the standard deviation of the flamingos. In contrast, the average difference in CORTf between the two methods was closer to 0 in flamingos, and hence, there was a smaller difference than in ducks. However, overall, the average difference was very small. Regarding the 95% confidence interval, the results for Mallards were closer, with −1.44 to +1.12, in contrast to those for the Greater Flamingos (−1.61 to +1.36) (see Figure 3 and Figure 4). Considering the figures, there was no increasing or decreasing trend of the data regarding the mean CORTf of the two methods and their respective average differences. In the case of the flamingos, the values tended to scatter around the center line (mean difference; Figure 4). The interval of mean values was closer in Mallards than in Greater Flamingos. There were three individuals outside the limits in the BA-plot of Mallards (see Figure 3), most likely because of the large difference of the CORTf values between the two sampling methods of one individual, as already seen in the CCC graph (see Figure 1). The graph for the Greater Flamingos was similar. There were three individuals outside the limits and, additionally, one with a very high mean value, and a small difference between the methods was observed (see Figure 4). This point represented the results of the individual with the high CORTf levels in both methods.

In summary, comparing the CORTf levels with standard deviation, the average difference, and the limits, the agreement between the two techniques, namely feather plucking and cutting, was better in the Mallards. However, concerning the CCC value, a better correlation was found for flamingos, whereas the figures (see Figure 1 and Figure 2) showed equally good correlation in both species. Overall, the graphs of the BA-plot indicate good concordance between the two methods for both species. Additionally, it should be noted, regarding the CORTf levels, that the cut samples produced slightly higher average values. However, these differences were marginal and therefore negligible.

As described in the Results section, the Mann-Whitney U test yielded no significant difference between males and females. Considering the box plot, it appeared relatively symmetrical for the Mallards (see Figure 5); the whiskers had approximately the same length in both sexes. In male ducks, the minimum and maximum values were smaller than those in females. The median was exactly in the middle in females and deviated in males. Likewise, the box was larger in males, indicating greater dispersion in this group. In the group of Greater Flamingos, the whiskers were symmetrical, i.e., with an equal distance between minimum and maximum to the interquartile range, which correlated with 50% of the data (see Figure 6). However, the male flamingos produced smaller maximum and minimum values. The medians appeared central in both sexes. However, the box for the female flamingos was larger, indicating greater dispersion. In summary, sex did not appear to influence CORTf. In Greater Flamingos, a greater dispersion was observed in females, but in Mallards, dispersion was greater in males. In addition, in both species, females had, on average, higher maximum and minimum values. This result leaves room for interpretation. In a study with Common Redpolls, a lower plasma level of CORT was measured in males [37]. In another project, a correlation with body condition and plasma CORT levels was detected in female Snow Petrels but not in males [40]. Therefore, it may be necessary to consider including body mass as a variable in future studies with respect to body condition. In summary, if there were no influence of sex on CORTf level according to our study, there would be potential practical benefits. In some species, no clear sexual dimorphism is seen; this would normally require sex analysis via DNA. Considering the results of our study, a reliable comparison of CORTf levels without knowing the sex could be possible, with practical relevance to the finding of suitable subject groups unaffected by the distribution of sex within and to the comparison of CORTf values of individuals regardless of their sex. Furthermore, laboratories that perform DNA sexing from feathers require a freshly plucked feather, which would not be possible with our validated less-invasive method of feather cutting.

Drawing a comparison between the wild Mallards and the Mulard Ducks of a conventional poultry farm examined in our last study, the mean CORTf values were slightly higher (difference 0.25 resp. 0.11) in the Mulards in both methods (plucked: 1.75 pg/mm; cut: 1.77 pg/mm) [20]. This result could suggest that the farm birds might experience more stress than the wild birds, although the stress experienced in the wild (e.g., regarding survival, nutrition, reproduction) should not be underestimated. In addition, the possible impact of the hunting season on the bird’s stress level should not be forgotten when interpreting CORTf of wild mallards. There are risk assessment studies that have found an increase in flight initiation distance (FID) during the hunting season [59]. This should be kept in mind, especially in future studies when comparing wild birds with captive birds. In our case, the difference shown between Mallards and Mulard Ducks was not outstanding, most likely because of differences in age and duck species.

Regarding the juvenile wild flamingos in contrast to the adult zoo Greater Flamingos of a study by Reese et al., the zoo birds had a mean value of 11.46 pg/mm, with a maximum of 20.93 pg/mm and a minimum of 2.66 pg/mm [27]. In this study, only plucked feather samples were taken. Comparing them to the plucked samples of our juvenile individuals, they resulted in an 8.95 lower mean CORTf. However, it is not useful to directly compare the CORTf values of these groups due to the differences in age and living conditions. Interestingly, Reese et al. described that the CORTf results strongly varied between the zoological institutions (variance between institutions = 53.82%), whereas within the single populations, CORTf proved to be relatively consistent [27].

Due to the aspects of comparability of our results, it can be referred to as the ‘STRANGE’ framework by M. Webster and C. Rutz, published in Nature in 2020 [60]. The authors note how large the variation is in the animal world, indicating the importance of building a framework to enable accurate comparison. To consider all these influences when studying individuals, the acronym STRANGE was invented, short for Social background; Trappability and self-selection; Rearing history; Acclimation and habituation; Natural changes in responsiveness; Genetic make-up; and Experience [60]. Taking this into account, a direct comparison between different bird species seems difficult, as well as a comparison between adults and juveniles. Therefore, to create representative studies, it is highly important to create data bases and include as accurate as possible descriptions of living conditions, husbandries, and behavior or internal factors (e.g., age, sex). Considering these influencing factors is the only way to correctly interpret CORTf as a stress measure.

## 5. Conclusions

In summary, the differences between the two sampling methods were negligible. Consequently, cutting feathers is a possible alternative technique. It should be added that this method, although it is less invasive, could be burdening for the individual, as living birds must still be caught and held. Hence, we suggest that such projects should be linked with capture events for different purposes. Only then, we act in the sense of animal welfare and refinement. Furthermore, it should be recommended to include more potentially influencing factors, not only the sex, but also body mass.

Considering the results from this and from another study [20], feather cutting can be used as a sampling method for CORTf determination.

## Figures and Tables

**Table 1:** Overview of the measured results (total feather length, weight of feathers, and CORTf) of 46 (47) Mallard (*Anas platyrhynchos*) compared between sexes, with 22 females and 24 (25) males, and sampling method (plucked vs. cut); 9 to 10 feathers were used for analysis. The values in brackets indicate the results including the outlier.

Sex		Total Length of Feathers (in mm)		Weight of Feathers (in mg)		CORTf (in pg/mm)		CORTf (in pg/mg)	
		Plucked	Cut	Plucked	Cut	Plucked	Cut	Plucked	Cut
Female	Mean	386	384	81.5	84.7	1.49	1.76	7.18	8.07
	Max	400	400	104.3	105.2	2.62	4.37	12.12	19.21
	Min	362	355	57.1	57.3	0.84	0.85	3.73	3.89
Male	Mean	390	388	84.1	89.7	1.51	1.58 (1.84)	7.11	6.87 (7.96)
	Max	412	400	106.4	113.2	2.91	3.26 (8.10)	14.31	13.35 (34.29)
	Min	375	369	66.0	66.5	0.90	0.82	4.04	3.40
Total mean		388	386	82.8	87.3	1.50	1.66	7.14	7.44

**Table 2:** Overview of the measured results (total feather length, weight of feathers, and CORTf) of 46 Greater Flamingos (*Phoenicopterus roseus*) compared between sexes, with 26 females and 20 males, and sampling method (plucked vs. cut); 7 to 10 feathers were used for analysis.

Sex		Total Length of Feathers (in mm)		Weight of Feathers (in mg)		CORTf (in pg/mm)		CORTf (in pg/mg)	
		Plucked	Cut	Plucked	Cut	Plucked	Cut	Plucked	Cut
Female	Mean	368	373	63.9	70.7	2.56	2.70	14.95	14.41
	Max	393	395	76.8	83.4	6.29	7.22	35.28	35.61
	Min	236	333	39.4	56.6	1.26	1.29	6.98	5.83
Male	Mean	371	372	77.7	83.7	2.44	2.54	11.65	11.25
	Max	393	396	89.5	95.6	4.11	4.21	18.46	17.71
	Min	330	341	63.2	74.0	1.68	1.45	8.18	6.73
Total mean		370	372	69.9	76.4	2.51	2.63	13.52	13.04
